# Supplementary material for: A capture enzyme-linked immunosorbent assay for detection of mosquito salivary protein-specific immunoglobulin E
Source: PLoS Negl Trop Dis. 2025 Aug 28;19(8):e0013468. doi: 10.1371/journal.pntd.0013468 (PMC12410881; doi:10.1371/journal.pntd.0013468)
Supplement: S1 Table — (DOCX) [file pntd.0013468.s001.docx]

**S1 Table. AAEL000749-specific IgE titers in the sera of mosquito bite-positive and -negative populations using the capture ELISA.**

| Positive population ID^1^ | ΔOD450^2^ | AAEL000749-specific IgE^3^ |
| --- | --- | --- |
| #1 | 0.058 | + |
| #2 | 0.057 | + |
| #3 | 0.023 | + |
| #4 | 0.013 | + |
| #5 | 0.015 | + |
| #6 | 0.016 | + |
| #7 | 0.028 | + |
| #8 | 0.045 | + |
| #9 | 0.028 | + |
| #10 | 0.0093 | + |
| #11 | 0.070 | + |
| #12 | 0.010 | + |
| #13 | 0.53 | + |
| #14 | 0.053 | + |
| #15 | 0.050 | + |
| #16 | 0.098 | + |
| #17 | 0.026 | + |
| #18 | 0.0070 | + |
| #19 | 0.16 | + |
| #20 | 0.027 | + |
| Negative Population ID^4^ | ΔOD450 | AAEL000749-IgE |
| #1 | 0.00066 | - |
| #2 | -0.0083 | - |
| #3 | -0.0026 | - |
| #4 | -0.0021 | - |
| #5 | 0.00016 | - |
| #6 | 0.0014 | - |
| #7 | -0.010 | - |
| #8 | -0.0037 | - |
| #9 | 0.0041 | - |
| #10 | -0.0072 | - |
| #11 | 0.0041 | - |
| #12 | -0.0093 | - |
| #13 | -0.0093 | - |
| #14 | 0.00066 | - |
| #15 | -0.0060 | - |
| #16 | -0.0093 | - |
| #17 | 0.0020 | - |
| #18 | -0.0085 | - |
| #19 | -0.0090 | - |
| #20 | 0.00096 | - |

Note:

1. The *Aedes aegypti* bite-positive population consists of severe dengue (SD) patients we recruited in Xishuangbanna, China, in August 2024. In August, the population density of the mosquitoes in Xishuangbanna reaches its annual peak, and the intensity of dengue transmission is also among the highest of the year. We enrolled 20 SD patients during August, collected their acute-phase sera, and measured the titers of AAEL000749-specific IgE in their sera using our capture ELISA. In Xishuangbanna, dengue virus is primarily transmitted by *Aedes aegypti* bites; therefore, the SD patients we recruited were theoretically repeatedly bitten by the mosquitoes (all volunteers self-reported a history of frequent mosquito bites before illness onset). Their acute-phase sera are thus theoretically expected to contain AAEL000749-specific IgE antibodies, and thus they can be considered an *Aedes aegypti* bite-positive population.

2. ΔOD450 is calculated as the OD450 value of the test serum minus the mean OD450 value of negative control sera.

3. The detection of AAEL000749-specific IgE was performed using our capture ELISA. The cut-off value for determining positivity of AAEL000749-specific IgE antibodies is set at two times the maximum ΔOD450 value of negative control sera (NC_max_), i.e., 2 × NC_max_. In this experiment, the cut-off value was 0.0047.

4. The *Aedes aegypti* bite-negative population consists of 20 volunteers recruited at Tsinghua University from December 2024 to February 2025, who have been living long-term in northern China, where *Aedes aegypti* is not distributed. Theoretically, these individuals have never been bitten by *Aedes aegypti*, and their sera are therefore expected to lack AAEL000749-specific IgE antibodies. Thus, they serve as the *Aedes aegypti* bite-negative population.
