# Supplementary material for: A capture enzyme-linked immunosorbent assay for detection of mosquito salivary protein-specific immunoglobulin E
Source: PLoS Negl Trop Dis. 2025 Aug 28;19(8):e0013468. doi: 10.1371/journal.pntd.0013468 (PMC12410881; doi:10.1371/journal.pntd.0013468)
Supplement: S2 Table — (DOCX) [file pntd.0013468.s002.docx]

**S2 Table. AAEL000749-specific IgE titers and total IgE concentration in *Aedes aegypti* bite-positive pooled human serum.**

| Dilution | AAEL000749-specific IgE  (ΔOD450) | Total IgE  (ng/mL) |
| --- | --- | --- |
| Undiluted | N/A | 6993.41 |
| 1:5 | 0.096 | 1398.68 |
| 1:10 | 0.047 | 699.34 |
| 1:20 | 0.031 | 349.67 |
| 1:40 | 0.0096 | 174.84 |
| **1:80** | **0.0058** | **87.42** |
| 1:160 | 0.0024 | 43.71 |
| Cut-off | 0.0047 | 65.09 |
