# Supplementary material for: A capture enzyme-linked immunosorbent assay for detection of mosquito salivary protein-specific immunoglobulin E
Source: PLoS Negl Trop Dis. 2025 Aug 28;19(8):e0013468. doi: 10.1371/journal.pntd.0013468 (PMC12410881; doi:10.1371/journal.pntd.0013468)
Supplement: Attachment — Submitted filename: Comments-PloS NTD-by Prof An.docx [file pntd.0013468.s003.docx]

Repeated exposure to mosquitoes can elicit allergic reactions, with mosquito salivary proteins (MSPs) and the corresponding IgE antibodies playing integral roles. Current ELISA and immunoblotting techniques for detecting MSP-specific IgE often face challenges due to interference from abundant, high-affinity IgG, which can significantly compromise sensitivity.

In this manuscript, Wang *et al.* present an innovative and highly efficient capture ELISA designed to address these limitations. The assay utilizes microplates coated with a monoclonal antibody targeting the IgE Fc region, thereby enriching specifically for IgE without affecting the antigen-binding F(ab)_2_ region. Instead of natural MSPs, recombinant MSPs expressed in *Drosophila* S2 cells were employed, enabling the detection of IgE responses to individual MSPs. Each recombinant MSP includes a C-terminal V5 tag, facilitating detection with a V5-specific monoclonal antibody, which improves specificity and streamlines the overall assay workflow. Signal amplification was achieved through the use of an HRP-conjugated secondary antibody and an ultrasensitive TMB substrate. Overall, this capture ELISA offers a highly specific and sensitive method for the detection of MSP-specific IgE in human and mouse serum samples. Moreover, detecting other antibody types, such as IgM, IgE, or IgA, which typically have significantly lower affinity and concentrations compared to IgG, faces a common challenge: interference from abundant IgG that cannot be ignored. The capture ELISA method proposed by Wang *et al*. may also serve as a valuable reference for the detection of antigen-specific IgE, IgM, or IgA.

The manuscript is well written, and the experimental details are also clearly presented. I only have some minor issues below, and addressing these could further improve the paper:

1. Although the authors provided a detailed description of the data expression and statistical methods used in the Materials and Methods section, it would be better to also include this information in the corresponding figure legends.
2. Figure 1, provide the full names for MSP, TMB, oxTMB, and HRP in the figure caption. In Fig1B, it would be clearer to label the “capture antibody” as “IgE-capture antibody” to specify its target.
3. Figure 2B and 2C, change the legend label from “mosquitoes” to “Number of mosquitoes.” In Figure 2C, modify the X-axis label from “bite” to “bites.”
4. Figure 5B, label each segment of the pie chart with its specific percentage value.
