# Supplementary material for: A capture enzyme-linked immunosorbent assay for detection of mosquito salivary protein-specific immunoglobulin E
Source: PLoS Negl Trop Dis. 2025 Aug 28;19(8):e0013468. doi: 10.1371/journal.pntd.0013468 (PMC12410881; doi:10.1371/journal.pntd.0013468)
Supplement: Attachment — Submitted filename: Response to Reviewers-20250722.docx [file pntd.0013468.s004.docx]

**PNTD-D-25-00667
A capture enzyme-linked immunosorbent assay for detection of mosquito salivary protein-specific immunoglobulin E
PLOS Neglected Tropical Diseases**

Dear Dr. Cheng,

Thank you for submitting your manuscript to PLOS Neglected Tropical Diseases. After careful consideration, we feel that it has merit but does not fully meet PLOS Neglected Tropical Diseases's publication criteria as it currently stands. Therefore, we invite you to submit a revised version of the manuscript that addresses the points raised during the review process.

Please submit your revised manuscript within 60 days Aug 30 2025 11:59PM. If you will need more time than this to complete your revisions, please reply to this message or contact the journal office at [plosntds@plos.org](mailto:plosntds@plos.org). When you're ready to submit your revision, log on to https://www.editorialmanager.com/pntd/ and select the 'Submissions Needing Revision' folder to locate your manuscript file.

Please include the following items when submitting your revised manuscript:

* A rebuttal letter that responds to each point raised by the editor and reviewer(s). You should upload this letter as a separate file labeled 'Response to Reviewers'. This file does not need to include responses to any formatting updates and technical items listed in the 'Journal Requirements' section below.
* A marked-up copy of your manuscript that highlights changes made to the original version. You should upload this as a separate file labeled 'Revised Manuscript with Track Changes'.
* An unmarked version of your revised paper without tracked changes. You should upload this as a separate file labeled 'Manuscript'.

If you would like to make changes to your financial disclosure, competing interests statement, or data availability statement, please make these updates within the submission form at the time of resubmission. Guidelines for resubmitting your figure files are available below the reviewer comments at the end of this letter.

We look forward to receiving your revised manuscript.

Kind regards,

Adly M.M. Abd-Alla, Prof asso.
Section Editor
PLOS Neglected Tropical Diseases

Adly Abd-Alla
Section Editor
PLOS Neglected Tropical Diseases

Shaden Kamhawi
co-Editor-in-Chief
PLOS Neglected Tropical Diseases
orcid.org/0000-0003-4304-636XX

Paul Brindley
co-Editor-in-Chief
PLOS Neglected Tropical Diseases
orcid.org/0000-0003-1765-0002

**Response to editors:**

Dear Adly, Shaden, and Paul

Thank you very much for the prompt and professional handling of our manuscript (**PNTD-D-25-00667, "A capture enzyme-linked immunosorbent assay for detection of mosquito salivary protein-specific immunoglobulin E"**). We are also deeply grateful for the positive comments and valuable suggestions provided by all three reviewers (***Reviewer #1:*** *“Methods that facilitate the estimation of the mosquito bite-related IgE are important because this value can inform on potential allergic response if the bites repeat”;* ***Reviewer #2:*** *“Wang et al. present an innovative and highly efficient capture ELISA designed to address these limitations.” and “The manuscript is well written, and the experimental details are also clearly presented.”;* ***Reviewer #3:*** *“This research article presents a significant advancement in the detection of mosquito salivary protein (MSP)-specific IgE, addressing critical limitations of current methods. Overall, this is a very strong manuscript. The authors have developed a novel capture ELISA method that demonstrates superior sensitivity and specificity compared to traditional indirect ELISA. The work is well-designed, executed, and clearly presented.” and “The writing quality of the manuscript is excellent, demonstrating a high standard of formal scientific English”*).

We have carefully revised the manuscript according to all comments and suggestions from the reviewers. We believe these revisions have greatly improved the significance, applicability, and readability of our study. Please find below our point-by-point responses to the reviewers’ comments for your consideration.

Best regards,

Gong Cheng, PhD

School of Basic Medical Sciences, Tsinghua University

Email: [gongcheng@mail.tsinghua.edu.cn](mailto:gongcheng@mail.tsinghua.edu.cn)

**Response to Reviewers**

***Reviewer #1****:*

*In the present manuscript, the authors describe the construction of a capture ELISA assay for detection of IgE directed against mosquito salivary protein AAEL000749. For this purpose, they first produce the salivary protein in insect cells, and purify it via cobalt affinity chromatography. Then they use it for mouse immunization, to obtain the sera to demonstrate the specificity of the ELISA. Their proposed ELISA uses the therapeutic antibody Omalizumab for specific IgE capture, recombinant V5-labelled antigen as a bait, and finally anti-V5 tag antibody and secondary HRP-labelled antibody for detection. Indeed, anti-AAEL000749 -immunized mice sera are the only ones testing positive, when examined along with sera from adjuvant - only, BSA, or AAEL6347 (unrelated mosquito saliva protein)-immunized mice. They the perform animal experiment and expose mice to mosquito bites, and measure IgE in mouse serum 4 weeks post mosquito bites. With a greater number of mosquitos, and continuing exposure, also the IgE titer in serum increases. This specific IgE can only be detected by Capture ELISA, but less with Indirect ELISA (at least as presented here). Also, healthy human volunteers were exposed to mosquito bites in a similar way, and Capture ELISA shows more sensitive capture of the AAEL000749-specific IgE than the indirect assay. Finally, the authors measure the antigen-specific IgE in 60 sera, and estimate the reactivity in fold of negative control, and the sera appear to have a higher content of AAEL000749-IgE in the months August-October than in January-March.*

*Methods that facilitate the estimation of the mosquito bite-related IgE are important because this value can inform on potential allergic response if the bites repeat. However, there is a number of questions and issues that the authors should address:*

***Q1: The authors claim that the ELISA they present is of a higher sensitivity and specificity than other reported methods, but the side-to-side comparison with other methods is not really shown, and sensitivity or specificity (i.e. detection limit / number of false positives) are not shown or estimated.***

**A1:** Thank you for your comment. In fact, Figures 2 and 4 already provide a side-by-side comparison of the performance between the classical indirect ELISA and our newly established capture ELISA in detecting MSP-specific IgE. Furthermore, Figure 3B demonstrates the specificity of our capture ELISA. The results indicate that our capture ELISA possesses higher detection efficiency and excellent specificity compared to the classical indirect ELISA.

To further address this point, we quantitatively assessed the detection limit, detection sensitivity, false positive rate, and false negative rate of our capture ELISA when used to detect *Aedes aegypti* salivary protein AAEL000749-specific IgE in human serum samples. First, we recruited 20 positive volunteers who had been bitten by *Aedes aegypti* mosquitoes and 20 negative volunteers who had not been bitten by the mosquitoes (S1 Table). The *Aedes aegypti* bite-positive volunteers consists of 20 severe dengue (SD) patients recruited in Xishuangbanna, China, in August 2024. In August, the population density of the mosquitoes in Xishuangbanna usually reaches its annual peak, and the intensity of dengue transmission is also correspondingly among the highest of the year. In Xishuangbanna, dengue virus is primarily transmitted by *Aedes aegypti* bites; therefore, the SD patients were theoretically repeatedly bitten by the mosquitoes (all volunteers self-reported a history of frequent mosquito bites before illness onset). Their acute-phase sera are theoretically expected to contain AAEL000749-specific IgE antibodies, and thus they can be considered an *Aedes aegypti* bite-positive population. We collected their acute-phase sera, and measured the titers of AAEL000749-specific IgE in their sera using our capture ELISA.

The results showed that, among the 20 volunteers who were positive for *Aedes aegypti* bites, all 20 had positive serum AAEL000749-specific IgE, and none were negative, indicating a test sensitivity of 100% (20/20) and a false negative rate of 0% (0/20). Among the 20 negative volunteers, none had positive serum AAEL000749-specific IgE, and all 20 were negative, resulting in a test specificity of 100% (20/20) and a false positive rate of 0% (0/20) (S1 Table). These results demonstrate that the capture ELISA we established possesses good sensitivity and specificity.

In addition, we pooled the serum from all volunteers who were positive for *Aedes aegypti* bites and designated this as the “positive serum” which represents the average level in individuals who are positive for *Aedes aegypti* bites in the general population. We serially diluted this serum and used the capture ELISA to detect the titer of AAEL000749-specific IgE at different dilutions. The detection limit of our assay is defined as the highest dilution at which AAEL000749-specific IgE is still detected as positive before it turns negative in the next dilution. In practice, the capture ELISA we developed can only provide a relative quantification of AAEL000749-specific IgE, and its detection limit is difficult to express in terms of the absolute content of AAEL000749-specific IgE due to the lack of a standard. However, the total IgE concentration in serum corresponding to this detection limit can be easily measured. Thus, we used the total IgE content at the detection limit serum dilution (1:80, S2 Table) as a surrogate for the detection limit of AAEL000749-specific IgE, which was calculated to be 87.42 ng/mL, and the total IgE concentration in the positive serum was 6993.41 ng/mL (S2 Table). We hope this equivalent characterization of the detection limit may be helpful and acceptable.

The above content has been integrated into the Results section of the revised manuscript (Line 416–454).

***Q2: The authors claim that the recombinant antigens produced here should be superior to bacterial, but there is not really a direct comparison shown, using either any biophysical method, or control antibody reactivity, or biological activity.***

**A2:** We sincerely appreciate your insightful comments. Indeed, many mosquito salivary proteins undergo glycosylation modifications. For example, D7 proteins and apyrase, both highly abundant in mosquito saliva, are well-characterized glycoproteins (Phattanawiboon et al., Trop Biomed 2014; Sor-Suwan et al., Parasitol Res 2013; Grossman et al., Insect Mol Biol 1993; Montero-Solis et al., Insect Mol Biol 2004). These glycosylation modifications have been shown to play crucial roles in maintaining the biological activity of these proteins (Walski et al. Insect Biochem Mol Biol 2017).

However, prokaryotic expression systems inherently lack the machinery for glycosylation and other types of post-translational modifications, making it impossible for proteins expressed in these systems to undergo such modifications. As a result, the biological activity of proteins produced using prokaryotic systems may be significantly compromised. In contrast, Drosophila S2 cells possess the full complement of eukaryotic post-translational modification pathways. Therefore, mosquito salivary proteins expressed in S2 cells are structurally and functionally more similar, or even identical, to their native counterparts found in mosquito saliva.

***Q3: Labels of the Figures and Legend text are currently not sufficient for understanding (please see specific remarks below), and the value of their developed test is indeed difficult to judge until they provide more information.***

**A3:** Thank you for your valuable suggestions. We have carefully revised all the figures and legends according to the helpful comments from you and other reviewers to make them more self-explanatory. At the same time, we have also revised and refined the manuscript in accordance with the suggestions from you and the other reviewers. We hope that the revised version better highlights the significance and value of our study.

***Q4: The abstract describes the methodology – I propose the findings of the study are highlighted instead.***

**A4:** Thank you very much for your insightful suggestion. We have made significant revisions to the Abstract by reducing the description of methodological details and increasing the emphasis on the results of our study, thus better highlighting the findings and innovation of this work. Please refer to the Abstract of the revised manuscript (Line 20–Line 46).

***Q5: Several passages in the text repeat twice or three times, and I suggest that these redundancies are removed.***

**A5:** Thank you for this kind reminder, we have removed or rephrased the redundant content or expressions according to your kind suggestions.

**Reviewer1: *Please find below a list of remarks which I hope will be helpful.***

**Authors’ response:** We sincerely appreciate your valuable comments and suggestions. We have made corresponding revisions to each of your points, and the detailed changes are outlined below for your reference.

***Q6: Lines 27-37: “Current enzyme-linked immunosorbent assay (ELISA) and immunoblotting methods for detecting MSP specific IgE suffer from interference by abundant, high-affinity IgG, leading to low sensitivity.“ – there is no direct comparison with current methods shown in this manuscript.***

**A6:** Thank you for your comment. In fact, Figures 2 and 4 already provide a side-by-side comparison of the performance between the classical indirect ELISA and our newly established capture ELISA in detecting MSP-specific IgE. Furthermore, Figure 3B demonstrates the specificity of our capture ELISA. The results indicate that our capture ELISA possesses higher detection efficiency and excellent specificity compared to the classical indirect ELISA.

To further address this point, we quantitatively assessed the detection limit, detection sensitivity, false positive rate, and false negative rate of our capture ELISA when used to detect *Aedes aegypti* salivary protein AAEL000749-specific IgE in human serum samples. First, we recruited 20 positive volunteers who had been bitten by *Aedes aegypti* mosquitoes and 20 negative volunteers who had not been bitten by the mosquitoes (S1 Table). The *Aedes aegypti* bite-positive volunteers consists of 20 severe dengue (SD) patients recruited in Xishuangbanna, China, in August 2024. In August, the population density of the mosquitoes in Xishuangbanna usually reaches its annual peak, and the intensity of dengue transmission is also correspondingly among the highest of the year. In Xishuangbanna, dengue virus is primarily transmitted by *Aedes aegypti* bites; therefore, the SD patients were theoretically repeatedly bitten by the mosquitoes (all volunteers self-reported a history of frequent mosquito bites before illness onset). Their acute-phase sera are theoretically expected to contain AAEL000749-specific IgE antibodies, and thus they can be considered an *Aedes aegypti* bite-positive population. We collected their acute-phase sera, and measured the titers of AAEL000749-specific IgE in their sera using our capture ELISA.

The results showed that, among the 20 volunteers who were positive for *Aedes aegypti* bites, all 20 had positive serum AAEL000749-specific IgE, and none were negative, indicating a test sensitivity of 100% (20/20) and a false negative rate of 0% (0/20). Among the 20 negative volunteers, none had positive serum AAEL000749-specific IgE, and all 20 were negative, resulting in a test specificity of 100% (20/20) and a false positive rate of 0% (0/20) (S1 Table). These results demonstrate that the capture ELISA we established possesses good sensitivity and specificity.

In addition, we pooled the serum from all volunteers who were positive for *Aedes aegypti* bites and designated this as the “positive serum” which represents the average level in individuals who are positive for *Aedes aegypti* bites in the general population. We serially diluted this serum and used the capture ELISA to detect the titer of AAEL000749-specific IgE at different dilutions. The detection limit of our assay is defined as the highest dilution at which AAEL000749-specific IgE is still detected as positive before it turns negative in the next dilution. In practice, the capture ELISA we developed can only provide a relative quantification of AAEL000749-specific IgE, and its detection limit is difficult to express in terms of the absolute content of AAEL000749-specific IgE due to the lack of a standard. However, the total IgE concentration in serum corresponding to this detection limit can be easily measured. Thus, we used the total IgE content at the detection limit serum dilution (1:80, S2 Table) as a surrogate for the detection limit of AAEL000749-specific IgE, which was calculated to be 87.42 ng/mL, and the total IgE concentration in the positive serum was 6993.41 ng/mL (S2 Table). We hope this equivalent characterization of the detection limit may be helpful and acceptable.

The above content has been integrated into the Results section of the revised manuscript (please refer to Line 416–454).

***Q7: Line 89: “with nice detection sensitivity”- this is colloquial language, please reword to “high detection sensitivity”, or similar.***

**A7:** Thank you for correction, we have rephrased the description as “high detection sensitivity” in the revised manuscript (Line 96-97).

**Q8: *Line 93: “specific IgG will preferentially bind to the embedded MSPs due to its superior content and affinity”. This sentence would be better with another word-order:*** ***immobilized MSPs will preferentially be occupied by the specific IgG due to its superior concentration. “superior affinity” – can such statement be generalized and could you provide a reference?***

**A8:** We greatly appreciate your suggestion regarding this improved expression. We have incorporated this expression in the revised manuscript (Line 100).

Regarding the comparison of affinities between antigen-specific IgG and IgE, we reviewed numerous studies. We confirmed that antigen-specific IgG is indeed present at much higher concentrations in serum than antigen-specific IgE (Wu et al., Nat Rev Immunol 2014; Gould et al., Nat Rev Immunol 2008). Although many studies suggest that IgE generally undergoes less affinity maturation than IgG, which may result in lower affinity (Talay et al., Science 2012; Yang et al., Immunity 2012; Erazo et al., Immunity 2007; Gould et al., Trends in Immunology, 2015), there is currently no direct evidence showing that antigen-specific IgG consistently has higher affinity than antigen-specific IgE. Therefore, our previous statement asserting stronger affinity of IgG was inaccurate. The higher titers of IgG we observed are mainly due to its greater abundance in serum. Accordingly, we have removed this claim from our manuscript. We sincerely appreciate your thorough comments, which enabled us to correct this inaccuracy promptly.

***Q9: Line 97: “needs to collect” –*** ***requires the use of***

**A9:** Thank you for your meticulous correction. We have now addressed and corrected them accordingly (Line 104).

***Q10: Line 100: “used in the current literature” –*** ***described in the current literature***

**A10:** We have corrected this accordingly.

***Q11: Line 104: “MSPs expressed in Drosophila S2 cells…” – this is a very generalized statement, and no evidence for this claim is presented. Additionally, reference 14 only shows the results for recombinantly expressed Aed al 2, Aed al 13 and Aed al 14, and these should not be generalized as MSPs.***

**A11:** Thank you for your correction. To avoid a generalized statement, we have replaced “MSPs” with the two specific *Aedes aegypti* salivary proteins purified in this study, AAEL0006347 and AAEL0000749 (Line 112).

***Q12: Line 111: “The highly pure MSPs…„ this manuscript does not show this data.***

**A12：**As stated in **A11**, we have replaced "MSPs" with the specific *Aedes aegypti* salivary proteins AAEL0006347 and AAEL0000749, which were purified in this study, to avoid over-generalization or misunderstanding. In Fig. 3A(i), we evaluated the purity of AAEL0006347 and AAEL0000749 using SDS-PAGE followed by Coomassie Brilliant Blue staining. The results demonstrated that the purity of both proteins was very high, exceeding 95%.

***Q13: Line 181: purification procedure (buffers) and protein measurement and storage should be described briefly.***

**A13:** Thank you. In the revised manuscript, we have provided a brief description of the protein purification steps, purity assessment and identification, as well as storage methods. Please refer to Line 182–Line 211.

***Q14: Line 192: “goat anti-mouse IgE secondary antibody” – please use the RRID identificators for commercially acquired antibodies (and please throughout the manuscript).***

**A14:** Thank you for your suggestion. We have now listed all the commercial antibodies in this study according to the RRID identifiers.

***Q15: Line 268: Figure 2 B, C - the OD 450 is low here. What does OD 450 (1:10 dilution) on the y-axis mean, is this serum dilution? Figure legend (0, 1, 5 or 10 mosquitos) should be explained in the text form.***

**A15:** We apologize for not having clearly explained the meaning of OD450 (1:10 dilution) in the original manuscript. In Figure 2B and 2C, the OD450 value represents the difference between the OD450 value of tested murine serum samples diluted 1:10 and the mean OD450 value of negative control murine serum samples also diluted 1:10. Therefore, a more accurate description should be ΔOD450 (OD450_tested sample_ – OD450_mean of the negative control_). We have now clarified the definition of ΔOD450 for the tested samples in revised manuscript (Line 228–Line 234 in the Methods, Line 348-Line351 in the Fig 2 legend).

Compared to the abundant levels of IgG in serum, the total IgE content in host serum is inherently very low, and the levels of MSP-specific IgEs are even lower, which is well reflected in the relatively low ΔOD450 values observed (despite amplification of the signal using HRP-labeled secondary antibodies). This also represents the greatest challenge in detecting MSP-specific IgE. One optimization strategy is to dilute the tested sera as little as possible, for example at 1:2 or 1:5, to maximize the amount of total IgE captured and thereby increase the OD450 value for MSP-specific IgE. We have also discussed this limitation and the optimization strategy in the Discussion section of the article (Line 564-Line 566).

We have included relevant information regarding the number of mosquitoes used to bite the host in the Figure 2 legend (Line 351–Line 352).

***Q16: Line 290: “Purity and identity” – western blot is performed with an anti-V5 antibody, so it does not give information on the identity of the protein.***

**A16:** We greatly appreciate your careful and insightful comments. In our study, the full-length coding sequence of the mosquito salivary protein—including both the secretion signal peptide and mature protein region, but excluding the stop codon—was cloned into the pMT/V5-His A expression vector to construct the pMT/MSP/V5-His A plasmid. The integrity of the construct was confirmed by DNA sequencing. In this system, the MSP protein and the V5-His tag are expressed as a single open reading frame (ORF) with a single stop codon located downstream of the V5-His tag. Thus, all MSP proteins expressed from this construct are theoretically fused to the V5 tag. This design enables specific detection of the V5 tag to reliably identify recombinant MSP expression. It is also important to note that endogenous proteins expressed by the host cells do not possess a V5 tag. Furthermore, we confirmed the authenticity of MSP expression by ensuring that the molecular weight observed in Western blot analysis using V5-tag antibody corresponds with the predicted theoretical molecular weight.

***Q17: Figure 4: Is the value on Y axis the actual OD 450 – the values are low for ELISA. What does OD 450 (1:5 dilution) on the y-axis mean, is this serum dilution? How can the U-shape of the curve in 4B be explained – is it possible that the results would look different with another serum dilution?***

**A17:** We apologize for not having clearly explained the meaning of OD450 (1:5 dilution) in the original manuscript. In Figure 4B and 4C, the OD450 value on Y axis represents the difference between the OD450 value of tested human serum samples diluted 1:5 and the mean OD450 value of negative control human serum samples also diluted 1:5. Therefore, a more accurate description should be ΔOD450 (OD450_tested sample_ – OD450_mean of the negative control_). We have now clarified the definition of ΔOD450 for the tested serum samples in revised manuscript (Line 256–Line 270 in the Methods, and Line 410-Line 413 in Figure 4 legend).

Compared to the abundant levels of IgG in serum, the total IgE content in host serum is inherently very low, and the levels of MSP-specific IgEs are even lower, which is well reflected in the relatively low ΔOD450 values observed (despite amplification of the signal using HRP-labeled secondary antibodies). This also represents the greatest challenge in detecting MSP-specific IgE. One optimization strategy is to dilute the tested sera as little as possible, for example at 1:2 or 1:5, to maximize the amount of total IgE captured and thereby increase the OD450 value for MSP-specific IgE. We have also discussed this limitation and the optimization strategy in the Discussion section of the article (Line 564-Line 566).

We believe that the U-shaped curve observed in Figure 4B may be caused by interference from mosquito salivary protein-specific IgG (MSP-IgG). Firstly, conventional indirect ELISA cannot rule out the interference of MSP-IgG in the detection of MSP-IgE. After 1–3 weeks of mosquito bites, both MSP-IgG and MSP-IgE gradually begin to develop, with MSP-IgG typically produced earlier and in greater quantities than MSP-IgE. However, during this stage, the levels of both antibodies in serum remain low, so the interference of MSP-IgG in MSP-IgE detection is relatively minor. By 3–4 weeks after repeated mosquito exposure, MSP-IgG levels and titers become significantly higher than those of MSP-IgE, leading to pronounced interference in the detection of MSP-IgE. Because MSP-IgG has far greater concentration than MSP-IgE in serum, it competitively inhibits the binding of MSP-IgE to the MSP antigen coated on the ELISA plate, resulting in artifacts where the apparent levels of MSP-specific IgE paradoxically decrease with prolonged mosquito exposure. This phenomenon highlights an inherent limitation of conventional indirect ELISA in MSP-IgE detection.

We hypothesize that, within a certain range of serum dilutions, this U-shaped curve may persist for the same reasons observed at the 1:5 dilution. In fact, we assessed MSP-IgE detection at both 1:2 (Fig. R1A) and 1:20 (Fig. R1B) serum dilutions using conventional indirect ELISA, and the results showed that the U-shaped curves persisted, favoring our analysis.

***Q18: Line 363: This is also a very interesting example of the AAEL000749 activity:*** [***https://pmc.ncbi.nlm.nih.gov/articles/PMC5025043/***](https://pmc.ncbi.nlm.nih.gov/articles/PMC5025043/)***.***

**A18:** Thank you for providing this important paper, we are happy to cite this work.

***Q19: Line 350: NC-is negative control, but what was the negative control used here.***

**A19:** The negative control sera were collected from healthy volunteers (4 males and 4 females, aged 18–30 years; n = 8) recruited at Tsinghua University. All participants have resided long-term in northern China, where *Aedes aegypti* is not present. Therefore, these individuals are theoretically unlikely to have ever been bitten by *Aedes aegypti*, and their sera are expected to lack IgE antibodies specific to *Aedes aegypti* MSPs. As such, these samples serve as appropriate negative control sera. This important information has been included in the revised manuscript (Line 258–Line 263 in the Methods, and Line 411-Line 412 in Figure 4 legend).

***Q20: Line 370: Reference 25 uses different antigens, not all antigens used were similar in antibody induction for human and mice. Which data indicate that IgG produced as a response is of higher affinity?***

**A20:** Thank you for this important correction. In Reference 25, the authors’ said “Due to the large amount of specific IgG in mouse serum which completely inhibits the binding of the small amount of specific IgE to the antigens coated on the ELISA microplates, serum specific IgE cannot be measured in mice by using a conventional ELISA.”

There are relatively few studies investigating mouse allergy models induced by mosquito bites or measuring MSP-specific IgE. Reference 25 happens to address both the mosquito allergy model and serum IgE detection, so we used this study as a reference. However, we overlooked an important detail: the original publication only clearly states that IgG levels are higher than IgE, but it does not explicitly compare the affinity between these two antibody isotypes. Therefore, in our revised version, we have removed the claim that MSP-specific IgG has higher affinity than MSP-specific IgE.

Thank you again for this important correction.

***Q21: Line 371: “Currently, two primary methods are reported…“ – this sentence repeats from the introduction***

**A21**: Thank you for pointing out this issue. We have revised the relevant statement as “At present, the literature describes two primary approaches for detecting MSP-specific IgE” (Line 502- Line 503).

***Q22: Line 386: “However, the method still has space for improvement” – the novel method described here and the referenced one should be compared side-to-side to confirm this.***

**A22:** Thank you for your valuable comment. We have addressed this issue in our responses **A1** and **A6** to **Q1** and **Q6**; please kindly refer to those sections for detailed explanations.

***Q23: Line 387: “collect a large amount of MSPs” - this sentence repeats from the introduction***

**A23:** We have now rephrased it as “The capture ELISA also necessitates gathering a substantial quantity of MSPs” (Line 517- Line 518)

***Q24: Line 388: “serum prepared from mosquito bitten rabbit may not be so specific or efficient” – why would other mosquito-bitten species produce serum that is more specific or efficient?***

**A24**: In Line 388, we wrote that “the anti-MSPs serum prepared from mosquito bitten rabbit may not be so specific or efficient for the detection of MSPs.” What we meant is that the antiserum generated from mosquito-bitten rabbits, as used in Reference 15, may be less specific and efficient for detecting MSPs compared to commercially available V5-tag monoclonal antibodies (since the recombinant MSPs carry a V5 tag, allowing detection with these commercial antibodies). To avoid misunderstanding, we have rephrased the sentence as: “the anti-MSPs serum prepared from mosquito bitten rabbit may not be as specific or efficient as a V5-tag monoclonal antibody for the detection of MSPs” (Lines 519–520).

***Q25: Line 405: “selected IgE specific to AAEL000749“ – the specificity should be demonstrated.***

**A25:** We have demonstrated the specificity of the capture ELISA for detecting AAEL000749-specific IgE in Figure 3B, along with the corresponding description in Lines 356 to 364. We believe this evidence is sufficient to establish the assay’s specificity. In addition, we have provided new experimental results that further demonstrate the specificity of our established capture ELISA. For details, please refer to Question **Q1** and our response **A1**.

***Q26: Line 419: for detection of MSP-specific***

**A26:** Thank you, it has been corrected now.

**Reviewer #2:**

*Repeated exposure to mosquitoes can elicit allergic reactions, with mosquito salivary proteins (MSPs) and the corresponding IgE antibodies playing integral roles. Current ELISA and immunoblotting techniques for detecting MSP-specific IgE often face challenges due to interference from abundant, high-affinity IgG, which can significantly compromise sensitivity.*

*In this manuscript, Wang et al. present an innovative and highly efficient capture ELISA designed to address these limitations. The assay utilizes microplates coated with a monoclonal antibody targeting the IgE Fc region, thereby enriching specifically for IgE without affecting the antigen-binding F(ab)2 region. Instead of natural MSPs, recombinant MSPs expressed in Drosophila S2 cells were employed, enabling the detection of IgE responses to individual MSPs. Each recombinant MSP includes a C-terminal V5 tag, facilitating detection with a V5-specific monoclonal antibody, which improves specificity and streamlines the overall assay workflow. Signal amplification was achieved through the use of an HRP-conjugated secondary antibody and an ultrasensitive TMB substrate. Overall, this capture ELISA offers a highly specific and sensitive method for the detection of MSP-specific IgE in human and mouse serum samples. Moreover, detecting other antibody types, such as IgM, IgE, or IgA, which typically have significantly lower affinity and concentrations compared to IgG, faces a common challenge: interference from abundant IgG that cannot be ignored. The capture ELISA method proposed by Wang et al. may also serve as a valuable reference for the detection of antigen-specific IgE, IgM, or IgA.*

*The manuscript is well written, and the experimental details are also clearly presented. I only have some minor issues below, and addressing these could further improve the paper:*

***Q1: Although the authors provided a detailed description of the data expression and statistical methods used in the Materials and Methods section, it would be better to also include this information in the corresponding figure legends.***

**A1:** Thank you for this nice suggestion, we have now included the data expression and statistical statements in corresponding figure legends.

***Q2: Figure 1, provide the full names for MSP, TMB, oxTMB, and HRP in the figure caption. In Fig1B, it would be clearer to label the “capture antibody” as “IgE-capture antibody” to specify its target.***

**A2:** Thank you for reminder. We have now provided the full names for these abbreviations, and changed “capture antibody” as “IgE-capture antibody”.

***Q3: Figure 2B and 2C, change the legend label from “mosquitoes” to “Number of mosquitoes.” In Figure 2C, modify the X-axis label from “bite” to “bites.”***

**A3:** We greatly appreciate your detailed corrections. In the revised manuscript, we have addressed these points; please refer to Figure 2B and 2C for details.

***Q4: Figure 5B, label each segment of the pie chart with its specific percentage value.***

**A4:** We now have labeled the pie charts with specific percentage values. Please refer to Figure 5B

**Reviewer #3:**

*Dear Editor and authors,

Thank you for the opportunity to review the manuscript titled "A capture enzyme-linked immunosorbent assay for detection of mosquito salivary protein-specific immunoglobulin E" (PNTD-D-25-00667).* *This research article presents a significant advancement in the detection of mosquito salivary protein (MSP)-specific IgE, addressing critical limitations of current methods. Overall, this is a very strong manuscript. The authors have developed a novel capture ELISA method that demonstrates superior sensitivity and specificity compared to traditional indirect ELISA. The work is well-designed, executed, and clearly presented. The findings have important implications for understanding mosquito allergy and for epidemiological surveillance.* *The writing quality of the manuscript is excellent, demonstrating a high standard of formal scientific English. However, I have identified a few areas where minor improvements could further enhance its clarity and completeness:*

**Authors’ response:**

Thank you very much for taking the time to review our manuscript and for your highly positive evaluation of our work, which is truly encouraging for us. We also greatly appreciate your valuable suggestions, which have been instrumental in further improving the quality and readability of our manuscript. We have revised the manuscript according to your comments, and the detailed responses to your suggestions are provided below for your kind review.

***Q1: In the abstract, the phrase "allowing detection with a V5-specific monoclonal antibody, enhancing specificity and" is repeated. Consider rephrasing for better conciseness. For example, the sentence could be streamlined to: "Each recombinant MSP carried a C-terminal V5 tag, enabling detection with a V5-specific monoclonal antibody, thereby enhancing specificity and simplifying the assay."***

**A1:** Thank you for your thoughtful correction. We are happy to accept this excellent suggestion.

***Q2: The discussion effectively highlights the advantages of the developed capture ELISA over existing methods. However, a dedicated, explicit paragraph or sentence discussing any inherent limitations of this specific capture ELISA would strengthen the manuscript. For instance, consider addressing aspects such as: Potential for cross-reactivity with other IgE types, if any, despite the Fc region capture; Cost-effectiveness or throughput considerations for very large-scale screening, if applicable; and Any specific challenges encountered during its development or application that were successfully overcome but are worth noting for future researchers.***

**A2:** Thank you for this important reminder and your helpful suggestions. We have now included a dedicated paragraph discussing the limitations and their optimization strategies of this capture ELISA, including potential cross-reactivity with other IgEs, relatively low ΔOD450 values, and the more complex procedures required compared to conventional indirect ELISA. Please refer to lines 550-572 for your review.

***Q3: The concluding sentence in the discussion, "In summary, the novel capture ELISA is a powerful tool for detection MSP-specific IgE in both mouse and human serum and deserves well promoting," is concise. However, briefly expanding on what "deserves well promoting" entails could be beneficial. What are the next logical steps for this assay? For example, its potential application in: Clinical diagnostics for mosquito allergy; Broader epidemiological studies to map IgE sensitization in different populations; Assessment of vaccine efficacy against mosquito-borne diseases; and Further characterization of specific MSPs as biomarkers.***

**A3:** Thank you very much for your valuable suggestion. We have briefly expanded the concluding sentence to place particular emphasis on the excellent detection performance and specificity of this capture ELISA. We believe this assay merits further promotion for future applications, such as the identification of specific allergens in mosquito saliva, the diagnosis and prognosis of mosquito allergy, and epidemiological monitoring of mosquito exposure levels in different populations. Please refer to Lines 573 - 578.

***Q4: Consider using the STARD protocol to describe your findings.***

**A4:** Thank you very much for your professional advice. As we are currently in the process of applying for a patent for the capture ELISA method developed in this study, it may not be convenient for us to disclose our methodology in accordance with the STARD protocol at this time. Nevertheless, we are happy to use the STARD protocol to describe other works.

Thank you again for this valuable suggestion!

***Q5: Describe the coating buffer used in the immunoassays.***

**A5：**Thank you for this kind reminder. The coating buffer used in the immunoassay is a carbonate-bicarbonate buffer (pH 9.6). Its composition is 1.59 g Na₂CO₃, 2.93 g NaHCO₃, and deionized water to a total volume of 1 L. We have now included this information in the Methods (Lines 216-217, and Lines 241-242).
